# Psychometric Evaluation of the Modes of Health Information Acquisition, Sharing, and Use Questionnaire: Prospective Cross-Sectional Observational Study

**DOI:** 10.2196/44772

**Published:** 2023-09-11

**Authors:** Lenette M Jones, Ronald J Piscotty Jr, Stephen Sullivan, Beatriz Manzor Mitrzyk, Robert J Ploutz-Snyder, Bidisha Ghosh, Tiffany Veinot

**Affiliations:** 1 School of Nursing University of Michigan Ann Arbor, MI United States; 2 School of Nursing Oakland University Rochester, MI United States; 3 Center for Sexuality & Health Disparities University of Michigan Ann Arbor, MI United States; 4 Department of Clinical Pharmacy College of Pharmacy University of Michigan Ann Arbor, MI United States; 5 School of Information University of Michigan Ann Arbor, MI United States

**Keywords:** psychometric evaluation, health information behavior, construct validity, reliability, chronic illness, MHIASU, hypertension

## Abstract

**Background:**

Health information is a critical resource for individuals with health concerns and conditions, such as hypertension. Enhancing health information behaviors may help individuals to better manage chronic illness. The Modes of Health Information Acquisition, Sharing, and Use (MHIASU) is a 23-item questionnaire that measures how individuals with health risks or chronic illness acquire, share, and use health information. Yet this measure has not been psychometrically evaluated in a large national sample.

**Objective:**

The objective of this study was to evaluate the psychometric properties of the self-administered MHIASU in a large, diverse cohort of individuals living with a chronic illness.

**Methods:**

Sharing Information, a prospective, observational study, was launched in August 2018 and used social media campaigns to advertise to Black women. Individuals who were interested in participating clicked on the advertisements and were redirected to a Qualtrics eligibility screener. To meet eligibility criteria individuals had to self-identify as a Black woman, be diagnosed with hypertension by a health care provider, and live in the United States. A total of 320 Black women with hypertension successfully completed the eligibility screener and then completed a web-based version of the MHIASU questionnaire. We conducted a psychometric evaluation of the MHIASU using exploratory factor analysis. The evaluation included item review, construct validity, and reliability.

**Results:**

Construct validity was established using exploratory factor analysis with principal axis factoring. The analysis was constricted to the expected domains. Interitem correlations were examined for possible item extraction. There were no improvements in factor structure with the removal of items with high interitem correlation (n=3), so all items of the MHIASU were retained. As anticipated, the instrument was found to have 3 subscales: acquisition, sharing, and use. Reliability was high for all 3 subscales, as evidenced by Cronbach α scores of .81 (acquisition), .81 (sharing), and .93 (use). Factor 3 (use of health information) explained the maximum variance (74%).

**Conclusions:**

Construct validity and reliability of the web-based, self-administered MHIASU was demonstrated in a large national cohort of Black women with hypertension. Although this sample was highly educated and may have had higher digital literacy compared to other samples not recruited via social media, the population captured (Black women living with hypertension) are often underrepresented in research and are particularly vulnerable to this chronic condition. Future studies can use the MHIASU to examine health information behavior in other diverse populations managing health concerns and conditions.

## Introduction

### Background

Health information is a critical resource for those with chronic illnesses such as hypertension. A better understanding of health information behaviors, such as acquisition, sharing, and use would help scientists develop and deliver more effective interventions [1-4]. Supporting preferred health information behaviors among individuals would enhance the management of their chronic illness [5-10], which may reduce morbidity and mortality and increase quality of life. Yet scientists and health care providers must first be able to capture and reliably measure these important health information behaviors.

The MHIASU questionnaire was developed by Veinot et al [11] to measure HIV information behavior among men who have sex with men. This scale was based on a body of qualitative research highlighting the variability of health information behaviors among people facing health risks or chronic illnesses to make evidence-based decisions about health management [12,13]. The MHIASU assesses a frequent pattern of acquiring, sharing, and using health information [11]. Health information behavior involves how an individual interacts with health information; this encompasses all of the above behaviors [14]. In recent qualitative and quantitative studies, it has been shown that health information behavior is associated with participation in self-management behavior and feelings of altruism and—in the case of information sharing—helping others [7,10,15,16].

Further defining the reliability of the MHIASU scale could facilitate more effective measurement of a complex phenomenon across multiple studies. For example, investigators often conduct secondary analyses of the Health Information National Trends Survey data on cancer-related information seeking and the use of health technologies from a nationally representative sample of individuals in the United States [17]. The survey is publicly available but does not appear to have a specific scale embedded to systematically measure varied health information behaviors. Other scales evaluate health information seeking [18-20], sharing [3,21], and use [22,23], but most measure them independently or are tied to specific technologies.

Health information use is a process involving the application of information to a health-related problem or situation. Information use assists individuals in the self-management of chronic conditions [24] by facilitating self-management activities such as decision-making, problem-solving, assessing and developing plans, using resources effectively, gaining emotional comfort, and changing behavior [25,26]. In addition to the use of health information, seeking and sharing of information warrant additional examination. Health information seeking is an action to obtain information to address a specific need [27], while health information sharing often refers to an individual giving information to other lay persons about self-management strategies [14,28]. In fact, taking the steps to acquire and share blood pressure information may have health implications. For example, it has been shown that seeking health information was associated with lower blood pressure levels [6].

Initial testing yielded internal reliability for the scale of α=.80-.94 [29]. Additional study results in diverse populations with chronic disease, but in small cohorts, show that the MHIASU is a reliable tool (see Table 1). While these studies have reported satisfactory Cronbach α scores for the MHIASU, no studies have reported a psychometric evaluation of this tool. Due to the continued use of the MHIASU [6,7,30], it is imperative to examine the instrument’s validity. The purpose of the Sharing Information study was to evaluate the ability of the MHIASU to measure the anticipated factors (health information acquisition, sharing, and use) in a large diverse cohort. The evaluation includes a review of the MHIASU items, and tests of construct validity and reliability.

**Table 1:** Modes of Health Information Acquisition, Sharing, and Use questionnaire published reports.

Study	Sample description		Information behavior factors, α		
	Type of information	Target sample, n	Acquisition	Use	Sharing
Veinot et al (2013) [11]	HIV—men who have sex with men	194	.80	.94	N/A^a^
Meadowbrooke et al (2014) [31]	HIV—men who have sex with men	163	.77	.93	N/A
Jones et al (2018) [7]	High blood pressure—Black women	194	.84	.68	.70
Jones et al (2018) [6]	High blood pressure—Black women	147	.84	N/A	N/A
Jones et al (2019) [30]	High blood pressure—Black men and women	19	.70	.90	.70

^a^N/A: not applicable.

### Items

The MHIASU consists of 23 items designed to assess responses according to 3 subscales: acquisition, sharing, and use of health information (Table 2). It was developed to measure participants' interaction with health information related to their health risks and chronic conditions; the language used in this evaluation was specific to African American/Black (Black) women and high blood pressure. The first subscale (acquisition) measures how the participant found information about blood pressure self-management (8 items). The second subscale (sharing) measures how the participant shared information about blood pressure self-management (5 items). The third subscale (use) measures how the participant used blood pressure information to make decisions about self-management (10 items). Each item was constructed to correspond with one of the three areas of focus (acquisition, sharing, and use).

**Table 2:** 23-Item Modes of Health Information Acquisition, Sharing, and Use factor loadings using principal axis factoring.

		Factor 1 (acquisition), factor loading		Factor 2 (sharing), factor loading	Factor 3 (use), factor loading
**Health information acquisition**					
	Q1: I looked for information about high blood pressure with someone else.	0.6	0.2		0.2
	Q2: People gave me information about high blood pressure without me asking for it	0.5	0.04		0.1
	Q3: I accidentally found information about high blood pressure while I looked for information on other topics.	0.4	0.1		0.2
	Q4: I learned unexpected things about high blood pressure from the media (eg, when I watch television, listen to the radio, or read the newspaper or magazines).	0.6	0.1		0.2
	Q5: I learned unexpected things about high blood pressure when I talked to other people.	0.6	0.2		0.2
	Q6: I asked someone else to look for information for me about high blood pressure.	0.5	0.1		0.1
	Q7: I went to places where I think I will learn new things about high blood pressure by myself (eg, events, public lectures, or workshops).	0.6	0.5		0.1
	Q8: I went to places where I thought I would learn new things about high blood pressure with someone else (eg, events, public lectures, or workshops).	0.6	0.5		0.1
**Health information sharing**					
	Q9: I organized events during which high blood pressure was discussed.	0.2	0.5		0.04
	Q10: I gave documents, internet links, or emails on high blood pressure to other people.	0.2	0.6		0.2
	Q11: I shared recipes that I think are healthy or will help with managing their high blood pressure.	0.2	0.6		0.3
	Q12: I told others about ways to exercise to help them lower their blood pressure.	0.1	0.7		0.3
	Q13: I gave people encouragement about lowering their blood pressure or maintaining the pressure in a safe range.	0.2	0.6		0.3
**Health information use**					
	Q14: I used information to decide how to lower blood pressure.	0.2	0.3		0.7
	Q15: I used information to make plans on how to lower my blood pressure.	0.1	0.3		0.8
	Q16: I used information to figure out how to lower my blood pressure safely.	0.2	0.3		0.8
	Q17: I used information to evaluate my risk for another chronic illness (eg, diabetes) related to high blood pressure.	0.1	0.3		0.7
	Q18: I used information to help me decide whether to see a doctor, nurse, or other health care professional to help with managing my high blood pressure.	0.3	0.1		0.6
	Q19: I used information to help me monitor and track my high blood pressure at home.	0.2	0.1		0.7
	Q20: I used information to help me understand my blood pressure levels or my other test results.	0.2	0.03		0.7
	Q21: I used information to plan or make blood pressure-friendly meals.	0.1	0.4		0.6
	Q22: I used information to change my overall approach to maintaining my health.	0.1	0.3		0.7
	Q23: I used information to ask a health-care professional questions about high blood pressure or get a second opinion from another provider.	0.3	0.1		0.6

### Response Format and Administration

A self-report summated rating scale was chosen for measuring the frequency of health information acquisition, sharing, and use behaviors of the participants over the past 12 months. The inventory is rated on a 5-point Likert scale with the following responses: “never,” (1) “rarely,” (2) “sometimes,” (3) “often,” (4) and “very often (5).” Participants were asked to select the response that corresponded with their level of agreement with each of the 23 questions and statements. Two examples include: “I look for information on high blood pressure by myself” and “I used information to plan or make high blood pressure-friendly meals.” Scores were calculated by summing the items for each scale, with higher scores indicating more frequent engagement in information behaviors. Scores on each subscale range from minimum to maximum: acquisition (8-40), sharing (5-25), and use (10-50).

## Methods

### Ethics Approval

We conducted Sharing Information, a prospective, cross-sectional observational study, to evaluate the psychometric properties of the MHIASU in a large national sample of Black women with hypertension. This study was deemed exempt from review by the University of Michigan institutional review board (HUM00142076).

### Recruitment

Individuals responding to Facebook campaign advertisements from August 2018 to December 2018 were invited to complete an eligibility screener using Qualtrics [32] data collection. The screening survey consisted of 5 questions to determine if interested individuals met study inclusion criteria: self-identifying as a woman and Black, living in the United States, being 18 years or older, reporting a diagnosis of hypertension from a health care professional, and providing information about their current blood pressure levels. Individuals received a message that there was no incentive for participation in the study. If they chose to complete the survey, it would help refine the MHIASU measure and inform subsequent studies, which may benefit Black women with hypertension in the future. If eligible, individuals were directed to complete and confirm informed consent by clicking “I consent,” if they agreed to participate in the study. Participants were then directed to the Qualtrics survey that contained the MHIASU questionnaire.

### Sample

A total of 320 self-identified Black women living with hypertension completed the survey. Their mean age was 59.6 (SD 10.1) years, mean duration of hypertension diagnosis was 14.7 (SD 10.5) years, and mean self-reported systolic and diastolic blood pressures were 142.8 (SD 19.1) mm Hg and 84.4 (SD 13.0) mm Hg, respectively. The majority of participants (203/320, 63.5%) had at least an associate degree level of education.

### Survey

The web-based eligibility screener, consent form, and self-administered MHIASU were completed, on average, in about 19.6 (SD 28.8) minutes. Participants completed a separate Qualtrics link to receive their incentive so that their survey responses remained anonymous.

### Statistical Analysis

Data were analyzed using SAS (version 9.4; SAS Institute) [33]. A correlational analysis was used to examine the interitem associations. To examine the structure of the 23 items, we submitted the scale data to an exploratory principal axis factor analysis with varimax rotation in order to assess eigenvalues and how well the individual items loaded on the 3 anticipated factors. To determine the reliability of the constructs, Cronbach α values were calculated for each of the 3 subscales.

## Results

### Analysis of Interitem Correlations and Communalities

Interitem correlations were examined for possible item extraction. Items that had correlations ≥0.8 or at least 50% or more interitem correlations of <0.3 were considered for removal. No items had 50% or more interitem correlations of <0.3. Factor 1 (acquisition): no items had interitem correlations (*r*) greater than or equal to 0.8. Factor 2 (sharing): no items had interitem correlations greater than or equal to 0.8. Factor 3 (use): 3 items had interitem correlations greater than or equal to 0.8 (questions 15, 16, and 20—see Textbox 1 for question text). High interitem correlation may indicate redundancy. Therefore, the items were removed, individually and as a group, to examine their impact on the factor structure. There were no improvements in factor structure, so we retained all 3 items.

**Modes of health information acquisition, sharing, and use questionnaire.**

**Health information acquisition (n=8)**

Prompt: Please respond to each statement, keeping in mind the ways in which you have found information on high blood pressure in the past 12 months.

- I looked for information about high blood pressure with someone else.
- People gave me information about high blood pressure without me asking for it.
- I accidentally found information about high blood pressure while I looked for information on other topics.
- I learned unexpected things about high blood pressure from the media (eg, when I watch television, listen to the radio, or read the newspaper or magazines).
- I learned unexpected things about high blood pressure when I talked to other people.
- I asked someone else to look for information for me about high blood pressure.
- I went to places where I think I will learn new things about high blood pressure *by myself* (eg, events, public lectures, or workshops).
- I went to places where I thought I would learn new things about high blood pressure *with someone else* (eg, events, public lectures, or workshops).

**Health information sharing (n=5)**

Prompt: Please respond to each of the following statements thinking about ways you may have shared information on high blood pressure the past 12 months.

- I organized events during which high blood pressure is discussed.
- I gave documents, Internet links, or emails on high blood pressure to other people.
- I shared recipes that I think are healthy or will help with managing their high blood pressure with others.
- I told others about ways to exercise to help them lower their blood pressure.
- I gave people encouragement about lowering their blood pressure or maintaining their blood pressure in a safe range.

**Health information use (n=10)**

Prompt: Please respond to each statement thinking about ways you may have used information on high blood pressure in the past 12 months.

- I used information to decide how to lower blood pressure.
- I used information to make plans on how to lower my blood pressure.
- I used information to figure out how to lower my blood pressure safely.
- I used information to evaluate my risk for another chronic illness (diabetes) related to high blood pressure.
- I used information to help me decide whether to see a doctor, nurse, or other health care professional to help with managing my high blood pressure.
- I used information to help me monitor and track my blood pressure at home.
- I used information to help me understand my blood pressure levels or my other test results.
- I used information to plan or make blood pressure-friendly meals.
- I used information to change my overall approach to maintaining my health.
- I used information to ask a health care professional questions about high blood pressure or get a second opinion from another provider.

### Construct Validity of the MHIASU

An exploratory principal axis factor analysis was conducted restricting to the anticipated domains of acquisition, sharing, and use (Table 2). As expected, there were 3 factors with eigenvalues exceeding 1 (Table 3), explaining 100% of the common variance—as we constrained the analysis to the anticipated domains. Factor 1 (acquisition; 8 items) assessed participants’ seeking or acquiring health information about blood pressure control, with loadings ranging from 0.4 to 0.6. Factor 2 (sharing; 5 items) evaluated their sharing of health information about blood pressure control with others with loadings ranging from 0.5 to 0.7. Factor 3 (use; 10 items) pertained to using health information about blood pressure control to manage one’s own health, with loadings ranging from 0.6 to 0.8. Factor 3 loaded as the first factor in our analysis and explained the maximum variance (74%) but is described last in this report to be consistent with the chronology of the questions.

**Table 3:** Eigenvalues and proportion of variance in the varimax rotated solution.

Factor	Subscale	Eigenvalue	Proportion of variance (%)	Factor loadings
1	Health information acquisition	1.8	16	0.4-.06
2	Health information sharing	1.1	10	0.5-0.7
3	Health information use	8.4	74	0.6-0.8

### Reliability of the MHIASU

The proportion of variance accounted for these factors ranged from 10% to 74%, with eigenvalues ranging from 1.1 to 8.4 (Table 4). The internal consistency reliability was high for all 3 factors as evidenced by Cronbach α coefficients ranging from .81 to .93 (Table 4).

**Table 4:** Factors and internal consistency reliability.

Factor	Subscale	Number of items	Items	Cronbach α
1	Health information acquisition	8	1-8	.81
2	Health information sharing	5	9-13	.81
3	Health information use	10	14-23	.93

## Discussion

### Principal Findings

The results from this psychometric evaluation (the reliability, construct validity, and internal consistency) provide support for the MHIASU as an instrument to measure self-reported acquisition, sharing, and use of health information among diverse populations facing health risks or diagnosed with a chronic illness. Our findings support the use of the MHIASU in Black women with hypertension. Furthermore, since the MHIASU was originally evaluated in a sample of men who have sex with men to examine correlates of HIV testing, we believe that this survey instrument may be of use in a wide range of clinical and public health contexts.

Although each scale could be used independently, it is recommended that all 3 scales be used together to capture an overall assessment of how individuals with health risks find, share, and use information to self-manage their chronic conditions or reduce their risk of health complications. Similar to the manner in which the MHIASU was developed [12,13], in future studies, scientists would need to adapt items based on the participants’ chronic illness or health risk [15]. Investigators can conduct literature reviews and qualitative studies and use their findings to guide the adaptation of the MHIASU to their population and chronic illness of interest.

### Strengths

The MHIASU queries how frequently participants interact with health information, specifically how they acquire the information, how they share information with others, and how they use the information to guide decision-making. These behaviors have been associated with self-management behaviors such as outlining plans and finding emotional comfort in managing a chronic illness and helping others [6-9,15]. Researchers and practitioners can use the MHIASU to better understand how individuals interact with health information, which may help to determine which type of interventions that patients will best respond to. These findings also establish a foundation for future examination of the validity and reliability of the MHIASU and further development of instruments exploring health information behavior among diverse demographic groups with various health concerns and chronic illnesses.

### Comparison to Prior Work

There are several scales and surveys that capture other aspects of health information behaviors [3,17-23]. Some of the scales focus on intentional information seeking [1,4,19,20] but do not offer the full range of behaviors captured in the MHIASU, such as unexpected health information finding, in addition to sharing and use. Other measures capture health information behaviors but are tied to specific technologies that frequently change, like Facebook [19], chat systems [21], or the internet [3,17]. One of the benefits of MHIASU is that it is independent of a specific technology. Lastly, the findings of this psychometric evaluation support the construct validity and reliability of each scale of the MHIASU questionnaire, while other surveys [17] do not appear to have valid, reliable scales embedded that capture health information behaviors.

### Limitations

Several limitations should be kept in mind when interpreting the results of this study. First, the sample was recruited from a social media site, which suggests that they may have relatively high digital literacy. Those with higher digital literacy may be more active seekers and users of information. Second, the women-only sample had relatively high levels of education; both factors may be associated with the frequency of information seeking [6,20]. While these characteristics are a solid basis from which to assess information-seeking modalities, it is possible that psychometric properties of the scale may differ in samples shown to seek information less often; therefore, there is a need for subsequent research to examine psychometric properties of the scale in such populations. Third, the responses “never” to “very often” were not defined for participants; therefore, these responses were subjective. Finally, we asked participants to think about their health information behaviors over the past year, which is a potential for recall bias. Nevertheless, the study was conducted with a group that is often underrepresented in research—Black women and for a health issue of significant importance to this group—hypertension. It has also been successfully used in another underrepresented group and within an important clinical context for that group: men who have sex with men and HIV testing promotion.

### Conclusions

Few measures assess how individuals with chronic illness and health risks interact with health information. The MHIASU is an instrument that may be used among a variety of patients with different health risks or chronic illnesses and from diverse backgrounds. To date, this measure has been validated in men who have sex with men, as well as in Black women and men. Given its success in these groups, in addition to the positive findings outlined in this report, the MHIASU can be adapted for use with other diverse populations to examine ways in which they acquire, share, and use health information. Additional studies are needed to evaluate the use of the MHIASU in other diverse groups, with other health concerns and conditions.

## Abbreviations

MHIASU: Modes of Health Information Acquisition, Sharing, and Use

## Data Availability

The data sets generated during this study are not publicly available.
